# Foot self-care knowledge and behaviors among adults with diabetes mellitus: a cross-sectional survey in Ras Al Khaimah, United Arab Emirates

**DOI:** 10.3389/fendo.2026.1930648

**Published:** 2026-09-17

**Authors:** Rose Ekama Ilesanmi, Rabab Gad Abd El-Kader, Rasha Babiker, Ramya Kundayi Ravi, Mohamed Anas Patni, Khaled Walid Elayyan, Meerah Ahmed Al-Ansi, Unique Great Efemu, Abdulla Hussain Karam, Ebrahim Ahmed Alshuweihi, Belal Mohammed Alsalem, Adeshina H. Adeniran, Rawya Ahmed Alshehi

**Affiliations:** 1 RAK College of Nursing, RAK Medical and Health Sciences University, Ras Al Khaimah, United Arab Emirates; 2 Faculty of Nursing, Mansoura University, Mansoura, Egypt; 3 RAK College of Medicine, RAK Medical and Health Sciences University, Ras Al Khaimah, United Arab Emirates; 4 RAK College of Pharmacy, RAK Medical and Health Sciences University, Ras Al Khaimah, United Arab Emirates; 5 Ras Al Khaimah Health Centre, Emirates Health Services, Ras Al Khaimah, United Arab Emirates; 6 Diabetes Centre, Ibrahim Bin Hamad Obaidullah Hospital, Emirates Health Services, Ras Al Khaimah, United Arab Emirates

**Keywords:** behaviors, diabetes mellitus, foot self-care, knowledge, United Arab Emirates

## Abstract

**Background:**

Diabetic foot complications are a major cause of morbidity, disability, and amputation worldwide, yet many complications are preventable through effective self-care. Limited evidence is available on foot self-care knowledge and behaviours among adults with diabetes in Ras Al Khaimah, United Arab Emirates.

**Aim:**

This study aimed to assess foot self-care knowledge and behaviours and identify factors associated with these outcomes among adults with diabetes mellitus in Ras Al Khaimah.

**Methods:**

A cross-sectional study was conducted among 384 adults with diabetes attending selected primary healthcare centres and the Diabetes Centre at Ibrahim Bin Obaidullah Hospital in Ras Al Khaimah. Participants were selected using convenience sampling. Data were collected using a structured validated questionnaire comprising an eight-item knowledge scale and the 29-item Nottingham Assessment of Functional Foot Care. Data were analysed using SPSS version 22. Descriptive statistics, chi-square tests, multivariable logistic and linear regression analyses.

**Results:**

Most participants had poor foot-care knowledge (77.3%). Regarding foot self-care behaviours, 69.3% reported moderate behaviour, 26.0% poor behaviour, and 4.7% good behaviour. Knowledge category was significantly associated with foot self-care behaviour category (χ² = 9.784, p = 0.008; Cramer’s V = 0.160). In multivariable logistic regression, females had lower odds of good knowledge than males (AOR = 0.45, 95% CI: 0.23–0.90, p = 0.023), and participants with type 2 diabetes had lower odds of good knowledge than those with type 1 diabetes (AOR = 0.43, 95% CI: 0.21–0.87, p = 0.020). In multivariable linear regression, female gender (B = 3.80, 95% CI: 1.99–5.61, p < 0.001), family history of diabetes (B = 1.89, 95% CI: 0.30–3.48, p = 0.020), and knowledge category (B = 1.83, 95% CI: 0.07–3.60, p = 0.041) were positively associated with foot self-care behaviour scores, while educational status (B = −1.57, 95% CI: −2.64 to −0.51, p = 0.004) and history of foot ulcer (B = −5.60, 95% CI: −7.81 to −3.38, p < 0.001) were negatively associated.

**Discussion:**

Gaps were identified in foot-care knowledge and self-care behaviours among participants. Factors associated with knowledge and foot self-care behaviours should be considered when developing targeted foot-care education and support for adults with diabetes in primary and secondary healthcare settings.

## 1 Introduction

Diabetes mellitus (DM) is a major public health challenge. Based on the International Diabetes Federation, an estimated 536.6 million adults aged 20–79 years were living with the condition in 2021. Projections suggest this number may reach around 783–853 million by 2045 (1). The disease imposes substantial morbidity through complications such as cardiovascular disease, kidney failure, and neuropathy, and generates enormous economic costs in healthcare and loss of productivity (1). Among these complications, diabetic foot disease, including ulcers and infection, is most disabling, accounting for the majority of non-traumatic lower-limb amputations globally (2). Preventive foot self-care behaviours and early injury detection can markedly reduce ulcer incidence by up to 50% and amputation risk by 85% when consistently practiced (3).

Regionally, the prevalence of diabetes in the Middle East and North Africa (MENA) region is among the highest globally, affecting approximately one in six adults. Specifically, in the Gulf Cooperation Council (GCC) countries comprising Bahrain, Kuwait, Oman, Qatar, Saudi Arabia, and the United Arab Emirates (UAE), a scoping review of the literature revealed a higher prevalence of 24%, compared with 16% in non-GCC countries (Lebanon, Cyprus, Iraq, Jordan, Palestine, Turkey, Syria, Egypt, Iran, and Yemen). The United Arab Emirates (UAE) exemplifies this trend, with adult diabetes prevalence reaching 19.5% in 2023 and forecasted to affect 1.6 million residents by 2031 (4, 5).

Foot care is a critical component of diabetes management, as it can prevent severe complications that may lead to infection and amputation. Patients who understand the risks associated with diabetic foot ulcers (DFUs) are better equipped to recognize early signs of foot injuries and take appropriate preventive measures (6, 7). This awareness includes identifying symptoms; selecting appropriate footwear; regularly cleaning and drying the feet, especially between the toes; applying moisturiser appropriately while avoiding application between the toes; inspecting the feet daily for cuts, blisters, or other injuries; trimming toenails straight across to prevent ingrown nails; wearing well-fitted and protective footwear; and avoiding walking barefoot (7).

Evidence from the literature within the region suggests that individuals with diabetes mellitus often exhibit significant gaps in their knowledge regarding foot self-care. For example, a study in Saudi Arabia in 2023 found that only 35% of patients with diabetes had good foot-care knowledge, of whom only 27% demonstrated good foot-care behaviors (8). Another study in Saudi Arabia among 385 patients showed moderate awareness (57%) and misconceptions, with only 16% demonstrating high knowledge and poor behavior (9). Similarly, studies in Dubai Emirate of the UAE revealed poor foot self-care behaviours.

To the best of the researchers’ knowledge, there is a paucity of studies conducted in the Emirate of Ras Al Khaimah among its diverse population, including Emiratis, South Asians, other Arab groups, and expatriate workers, who may experience unique occupational and healthcare-access-related risks. This evidence gap may contribute to the burden of diabetic foot complications in Ras Al Khaimah, where primary care services are overstretched and podiatry resources are limited, potentially increasing the likelihood of lower-limb amputation (10, 11).

In the absence of robust local data on patients’ foot self-care knowledge and behaviours, the development of targeted nursing interventions and context-specific policies remains constrained. Therefore, this study aimed to assess foot self-care knowledge and behaviours among adults living with diabetes mellitus in the Emirate of Ras Al Khaimah.

## 2 Methodology

### 2.1 Study design and setting

A descriptive cross-sectional study was conducted in the diabetes clinics of Ibrahim Bin Obaidullah Hospital (IBOH) and Al Kuwaiti Primary Health Centre in the Emirate of Ras Al Khaimah, United Arab Emirates. IBOH is a secondary-care hospital that provide specialized diabetes service via diabetic clinics and out-patient departments, including, routine diabetes follow-up, diabetes education, complication screening, and specialist referral. Beyond endocrine care, the hospital supports the community with an array of essential medical specialties, including an internationally accredited stroke and neurology unit, expanded intensive care and dialysis departments, cardiology, dermatology, and psychiatric care.

Al Kuwaiti Primary Health Centre is a primary healthcare facility providing comprehensive outpatient services, including screening, diagnosis, follow-up, and management of patients with diabetes. Both these hospitals receive patients through routine appointments, referrals and walk-in services. Data were collected over a three -month period from June to August 2025, following approval from the relevant research ethics committee. The study is reported in accordance with the STROBE guidelines.

### 2.2 Participants and eligibility criteria

Adults aged 18 years or older with a confirmed clinical diagnosis of diabetes mellitus for at least one year were eligible for inclusion, ensuring adequate duration of disease experience and exposure to diabetes self-management and foot-care practices. Patients younger than 18 years, those with bilateral lower-limb amputations, gestational diabetes, severe cognitive or visual impairment, severe acute illness, inability to communicate effectively, or psychiatric illness that could interfere with comprehension or accurate completion of the questionnaire were excluded. Eligible patients were recruited sequentially until the target sample size was achieved using a convenience sampling technique.

### 2.3 Sample size

The sample size was calculated using the Statulator online calculator for a single population proportion, assuming a 95% confidence level, a 5% margin of error, and a conservative prevalence estimate of 50% (12). After accounting for a 5% non-response rate, the minimum required sample size was 385 participants.

### 2.4 Measurement and data collection instruments

Data were collected using a structured interviewer-administered questionnaire comprising three sections.

Section 1: Sociodemographic and clinical characteristics consisted of items assessing age, sex, marital status, educational level, occupation, type and duration of diabetes, family history of diabetes, comorbidities, diabetes-related complications, history of foot ulcer, and type of diabetes medication. These items were developed by the investigators based on the study objectives and existing literature.

Section 2: Diabetes foot self-care behaviours comprised the 29-item revised Nottingham Assessment of Functional Footcare (NAFF) developed and validated by Lincoln et al. (13). The NAFF was adopted for the present study without modification to the item content or scoring system. Each item is scored on a four-point frequency scale from 0 to 3, with the scoring direction varying according to whether the reported behaviour represents protective or potentially harmful foot-care behaviour. Thus, higher scores consistently represent more appropriate foot-care behaviour. The total score is obtained by summing the 29 item scores, with a possible range of 0–87; higher scores indicate better foot-care practices (13). For descriptive and categorical analyses, total NAFF scores were converted to a percentage of the maximum possible score and categorized as poor (<50%), moderate (50.0–69.9%), or good (≥70.0%) foot self-care behaviour, corresponding to raw scores of 0–43, 44–60, and 61–87, respectively. The percentage cut-points were based on the approach used by Alsaleh et al. [14]. These categories were used for descriptive and categorical analyses; the continuous total NAFF score was retained for multivariable linear regression.The scale has demonstrated a Cronbach’s alpha of 0.73, indicating acceptable internal consistency.

Section 3: Foot-care knowledge comprised an eight-item knowledge questionnaire adopted from Aljaouni et al. (8) The questionnaire consisted of eight dichotomous yes/no items addressing awareness of diabetic foot ulcers, loss of protective sensation, the relationship between sensory loss and ulceration, impaired circulation and gangrene, and previous exposure to foot-care educational materials. Each item was scored as 1 for a correct response and 0 for an incorrect response, yielding a total knowledge score of 0–8. Scores of 0–5 were classified as poor knowledge and 6–8 as good knowledge (8). The scale has demonstrated a Cronbach’s alpha of 0.73, indicating acceptable internal consistency.

The English questionnaire was translated into Arabic using **a** forward–backward translation procedure. The initial translation was performed independently by a bilingual translator familiar with health terminology, followed by an independent back-translation into English by a second bilingual translator who was blinded to the original questionnaire. The original and back-translated versions were compared to identify discrepancies in semantic and conceptual meaning. An expert panel subsequently reviewed the Arabic version for linguistic clarity, cultural relevance, and conceptual equivalence. Necessary linguistic refinements were made before the final version was administered.

The questionnaire was prepared in English and translated into Arabic using forward and back-translation procedures. The questionnaire underwent content validation by seven subject experts from the field of medicine, nursing, endocrinology and public health yielding a Content Validity Index (CVI) of 0.91, and was subsequently pilot-tested among 10 patients to assess clarity and comprehensibility. The final questionnaire was administered in Arabic and English according to participants’ language preference.

### 2.5 Statistical analysis

Data were analysed using SPSS version 22. Descriptive statistics were used to summarize participant characteristics, with frequencies and percentages reported for categorical variables and means with standard deviations reported for continuous variables. The distribution of continuous variables was assessed using the Shapiro–Wilk test, supported by visual inspection of histograms and Q–Q plots. Parametric tests were applied when the assumptions of approximate normality, independence of observations, and, where applicable, homogeneity of variances were satisfied. Homogeneity of variances was assessed using Levene’s test. A one-sample t-test compared the mean foot care knowledge score with that reported in a previous Dubai-based study. Associations between categorical variables were assessed using Chi-square tests. Pearson’s correlation coefficient examined relationships between continuous variables, and simple linear regression was used to assess whether knowledge predicted foot care practices. Independent t-tests compared mean scores between groups where appropriate. Logistic regression analysis was conducted when foot care behaviour outcomes were dichotomized. Statistical significance was set at *p* < 0.05. Missing data were minimal and variable-specific. Duration of diabetes was missing for five participants (1.3%), while comorbidity and diabetes-related complications were each missing for one participant (0.3%). No imputation was performed. Descriptive analyses were based on available observations, and multivariable regression analyses used complete-case analysis, resulting in an analytic sample of 382 participants.

## 3 Results

### 3.1 Profile of participants

The study included 384 patients with a mean age of 42.86 ± 14.72 years (**Table 1A**). Most of them were male (64.3%) and married (57.0%). Nearly half had a college-level education (47.7%), and most were employed, predominantly as government employees (48.2%), followed by private employees (37.2%).

**Table 1A:** Demographic characteristics of study participants (N = 384).

Variable	Category	n	%
Age (years)	Mean ± SD	42.86 ± 14.72	—
Gender	Male	247	64.3
	Female	137	35.7
Marital Status	Single	109	28.4
	Married	219	57.0
	Widowed	31	8.1
	Divorced	25	6.5
Educational Status	Illiterate	12	3.1
	Primary	89	23.2
	High school	100	26.0
	College	183	47.7
Occupation	Government employee	185	48.2
	Private employee	143	37.2
	Student	56	14.6

Most of them reported to have Type 2 diabetes (66.7%). The duration of diabetes was most commonly between less than 10 years, with 32.8% having 6–10 years and 32.0% less than 5 years.

Most participants did not report comorbidities (64.1%) or complications (58.9%), and a large majority had no history of foot ulcers (82.3%). Regarding treatment, over half of the participants (50.5%) were using a combination of injections and oral medications (**Table 1B**).

**Table 1B:** Clinical characteristics of study participants (N = 384).

Variable	Category	n	%
Type of Diabetes	Type 1	112	29.2
	Type 2	256	66.7
	Not sure	16	4.2
Duration of Diabetes	<5 years	123	32.0
	6–10 years	126	32.8
	11–15 years	69	18.0
	>16 years	61	15.9
	Not reported	5	1.3
Prescence of Comorbidity	Yes	137	35.7
	No	246	64.1
	Not reported	1	0.3
Complications	Yes	157	40.9
	No	226	58.8
	Not reported	1	0.3
History of Foot Ulcer	Yes	68	17.7
	No	316	82.3
Type of Medications	Injections only	34	8.9
	Pills only	156	40.6
	Both injections and pills	194	50.5

### 3.2 Foot self-care knowledge and behaviours

Most of the patients found to have poor knowledge (77.3%), while only 22.7% had good knowledge(**Table 2A**). In terms of behaviour, most participants exhibited moderate behaviour (69.3%), followed by poor behaviour (26.0%), with only a small proportion demonstrating good behaviour (4.7%).The item-wise details of knowledge is given in **Figure 1**.

**Table 2A:** Distribution of participants by level of foot-care knowledge and diabetes foot self-care behaviour (N = 384).

Variables	Category	n	%
Knowledge	Poor	297	77.3
	Good	87	22.7
Behaviour	Poor	100	26.0
	Moderate	266	69.3
	Good	18	4.7

NAFF behaviour: poor = 0–43 (<50%); moderate = 44–60 (50.0–69.9%); good = 61–87 (≥70%).

**Figure 1:**
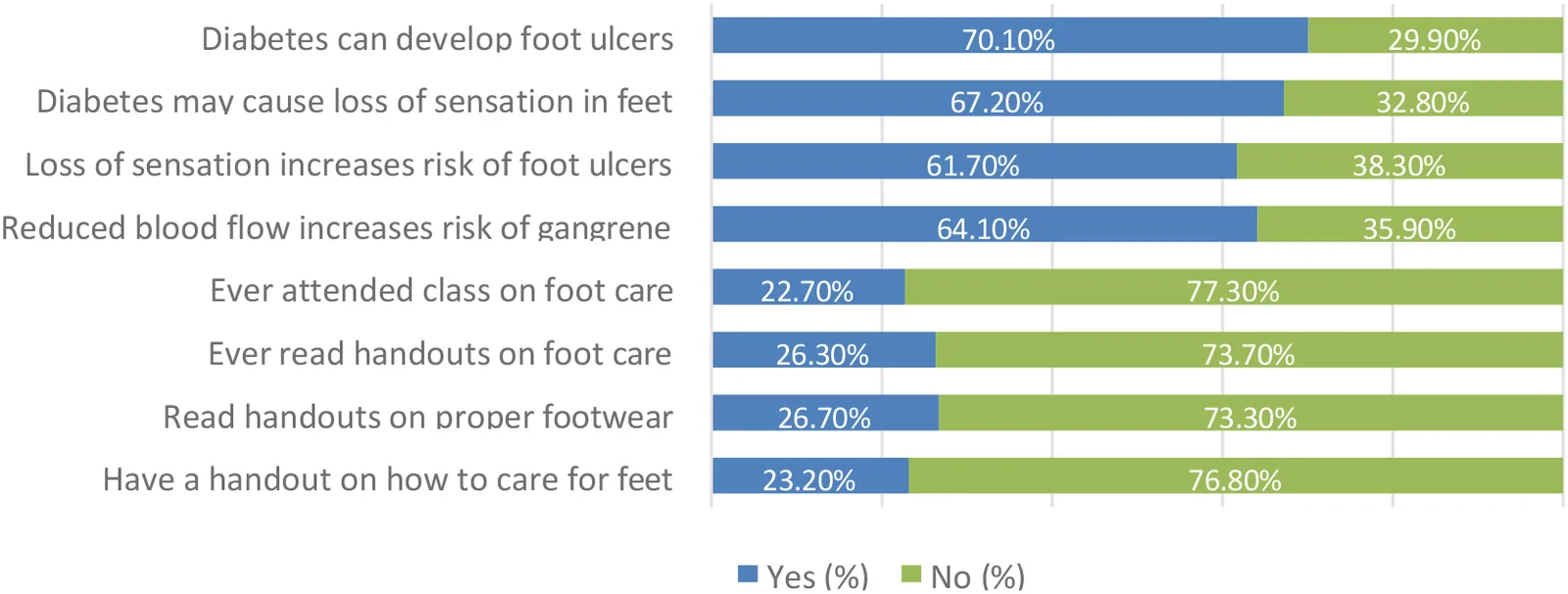
Distribution of participants’ responses (Yes/No) to individual foot-care knowledge items (N = 384).

A statistically significant association (**Table 2B**) was observed between knowledge category and behaviour category (χ² = 9.784, p = 0.008). Among participants with poor knowledge, 24.9% demonstrated poor behaviour, 72.1% moderate behaviour, and 3.0% good behaviour. In contrast, participants with good knowledge showed 10.3% good behaviour compared to 3.0% among those with poor knowledge, although moderate behaviour remained the most common category in both groups (59.8% vs 72.1%). The effect size was small (Cramer’s V = 0.160).

**Table 2B:** Association between foot-care knowledge category and self-care behaviour category among participants (N = 384).

Knowledge category	Poor behaviour n (%)	Moderate behaviour n (%)	Good behaviour n (%)	Total n (%)
Poor Knowledge (n=297)	74 (24.9%)	214 (72.1%)	9 (3.0%)	297 (100%)
Good Knowledge (n=87)	26 (29.9%)	52 (59.8%)	9 (10.3%)	87 (100%)
Total (N = 384)	100 (26.0%)	266 (69.3%)	18 (4.7%)	384 (100%)

Pearson Chi-square (χ²) = 9.784, df = 2, p = 0.008, Cramer’s V = 0.160.

### 3.3 K cluster analysis

K-means cluster analysis (**Table 3A**) identified three distinct behavioural profiles among participants.

**Table 3A:** Distribution of participants across foot-care behavioural clusters (N = 384).

Cluster	Frequency	Percentage
Cluster 1 – Low–Moderate Adherence	91	23.7
Cluster 2 – Low Adherence	172	44.8
Cluster 3 – High Adherence	121	31.5
Total	384	100

Cluster 2 (44.8%) represented a low-adherence group characterized by consistently low engagement in preventive foot-care practices. Cluster 3 (31.5%) demonstrated high adherence, including strong avoidance of barefoot walking and heat exposure. Cluster 1 (23.7%) showed mixed adherence, with moderate engagement in routine care but better avoidance of high-risk behaviours. Convergence was achieved after 10 iterations.

One-way ANOVA demonstrated (**Table 3B**) a significant difference in mean behaviour scores across the three clusters (F(2, 381) = 116.81, p < 0.001). Cluster 3 exhibited the highest mean behaviour score (55.07 ± 4.78), followed by Cluster 1 (47.42 ± 6.30), while Cluster 2 had the lowest mean behaviour score (44.37 ± 6.48). The effect size was large (η² = 0.380). *Post hoc* comparisons revealed statistically significant differences between all cluster pairs (p < 0.001).

**Table 3B:** Comparison of mean diabetes foot self-care behaviour scores across identified behavioural clusters (N = 384).

Cluster	Frequency	Mean behaviour score ± SD
Cluster 1 (Low–Moderate Adherence)	91	47.42 ± 6.30
Cluster 2 (Low Adherence)	172	44.37 ± 6.48
Cluster 3 (High Adherence)	121	55.07 ± 4.78
Total	384	48.47 7.54

One-way ANOVA: F(2, 381) = 116.81, p < 0.001; Effect size: η² = 0.380 (large); *Post hoc* (Bonferroni): Cluster 1 vs Cluster 2: p < 0.001, Cluster 1 vs Cluster 3: p < 0.001, Cluster 2 vs Cluster 3: p < 0.001.

A significant association (**Table 3C**) was observed between behavioural cluster membership and knowledge category (χ²(2) = 6.42, p = 0.040). Participants in Cluster 2 (low adherence) demonstrated the highest proportion of good knowledge (26.7%), whereas Cluster 1 showed the lowest proportion (13.2%). The effect size was small (Cramer’s V = 0.129).

**Table 3C:** Association between behavioural cluster membership and knowledge category (N= 384).

Cluster	Poor knowledge	Good knowledge	Total n
	Frequency (percentage)	Frequency (percentage)	Frequency (percentage)
Cluster 1 (Low–Moderate Adherence)	79 (86.8%)	12 (13.2%)	91 (23.7%)
Cluster 2 (Low Adherence)	126 (73.3%)	46 (26.7%)	172 (44.8%)
Cluster 3 (High Adherence)	92 (76.0%)	29 (24.0%)	121 (31.5%)
Total	297 (77.3%)	87 (22.7%)	384 (100%)

Pearson Chi-square (χ²) = 6.42, df = 2, p = 0.040; Cramer’s V = 0.129.

Multivariable logistic regression (**Tables 4**, **5**)was conducted to identify predictors of higher foot-care knowledge. The overall model was statistically significant (χ²(18) = 35.845, p = .007) and demonstrated good calibration (Hosmer–Lemeshow p = .964). The model explained 13.7% of the variance in knowledge level (Nagelkerke R² = .137). 217.

**Table 4:** Multivariable logistic regression analysis of factors associated with good foot-care knowledge among participants (n = 384).

Predictor	Category (vs reference)	B	AOR (Exp(B))	95% CI	p-value
Age	Continuous	0.004	1.00	0.98 – 1.03	.766
Gender	Female vs Male (Reference)	-0.798	0.45	0.23 – 0.90	.023*
Marital Status	(Overall)	—	—	—	.358
	Married vs Single (Reference)	0.394	1.48	0.67 – 3.31	.336
	Widowed vs Single (Reference)	-0.937	0.39	0.07 – 2.11	.275
	Divorced vs Single (Reference)	0.029	1.03	0.27 – 3.96	.966
Educational Status	(Overall)	—	—	—	.033*
	Primary vs Illiterate (Reference)	-0.782	0.46	0.08 – 2.70	.388
	High school vs Illiterate (Reference)	0.182	1.20	0.21 – 7.02	.840
	College vs Illiterate (Reference)	0.667	1.95	0.32 – 11.71	.466
Type of Diabetes	(Overall)	—	—	—	.048*
	(1) vs Ref	-0.857	0.43	0.21 –0.87	.020*
	(2) vs Ref	-1.359	0.26	0.04 – 1.51	.132
Occupation	(Overall)	—	—	—	
	Private employee vs Govt. employee (Reference)	0.407	1.50	0.79 – 2.84	.211
	Student vs Govt. employee (Reference)	0.719	2.05	0.69 – 6.12	.197
Family History of Diabetes	Yes vs No	-0.218	0.80	0.45 – 1.44	.463
Comorbidity	Yes vs No	0.321	1.38	0.72 – 2.63	.328
Complications	Yes vs No	-0.036	0.96	0.47 – 1.97	.921
History of Foot Ulcer	Yes vs No	0.628	1.87	0.81 – 4.33	.142
Type of Medications	(Overall)	—	—	—	.612
	0.407	1.50	0.79 – 2.84	.211	
	0.719	2.05	0.69 – 6.12	.197	
	Pills only vs Injections only (Reference)	0.369	1.45	0.49 – 4.26	.503
	Both injection and pills vs Injections only (Reference)	0.015	1.02	0.36 – 2.83	.977

Omnibus χ²(18) = 35.845, p = .007; Nagelkerke R² = 0.137; Hosmer–Lemeshow χ²(8) = 2.460, p = .964; *Statistically significant at p < 0.05.

**Table 5:** Multiple linear regression analysis of factors predicting diabetes foot self-care behaviour score among participants (n = 384).

Predictor	B	SE	Beta	95% CI	p
Age	0.050	0.037	.098	-0.021 – 0.122	.169
Gender	3.798	0.921	.242	1.986 – 5.609	<.001*
Marital status	-0.807	0.555	-.084	-1.899 – 0.285	.147
Educational status	-1.573	0.543	-.187	-2.640 – -0.505	.004*
Occupation	-0.748	0.678	-.071	-2.081 – 0.586	.271
Type of Diabetes	-0.816	0.763	-.056	-2.316 – 0.685	.286
Family history	1.888	0.810	.119	0.296 – 3.481	.020*
Comorbidity	-0.822	0.882	-.052	-2.556 – 0.911	.352
Complications	-1.057	0.955	-.069	-2.935 – 0.821	.269
History of foot ulcer	-5.596	1.127	-.284	-7.812 – -3.380	<.001*
Type of medications	1.125	0.676	.096	-0.205 – 2.454	.097
Knowledge	1.834	0.896	.102	0.073 – 3.596	.041*

F(12, 369) = 5.732, p <.001); R² = 0.157; adjusted R² = 0.130; *Statistically significant at p < 0.05.

Females had significantly lower odds of good foot-care knowledge compared with males (AOR = 0.45, 95% CI: 0.23–0.90, p = .023; **Table 4**). Educational status was significantly associated with knowledge at the overall variable level (p = .033), although none of the individual educational categories differed significantly from the reference category. Type of diabetes was also significantly associated with knowledge overall (p = .048). Participants with type 2 diabetes had lower odds of good knowledge compared with those with type 1 diabetes (AOR = 0.43, 95% CI: 0.21–0.87, p = .020), whereas the comparison for participants unsure of their diabetes type was not statistically significant (AOR = 0.26, 95% CI: 0.04–1.51, p = .132). Occupation was not significantly associated with knowledge (overall p = .337). No statistically significant associations were observed for age, marital status, family history of diabetes, comorbidity, complications, history of foot ulcer, or type of medications.

Multiple linear regression analysis was performed to identify predictors of foot-care behaviour score.

The overall model was statistically significant (F(12, 369) = 5.732, p <.001) and explained 15.7% of the variance in behaviour score (R² = 0.157; adjusted R² = 0.130).

Gender was a significant predictor of behaviour, with females demonstrating higher behaviour scores compared to males (B = 3.80, p <.001). Educational status was negatively associated with behaviour (B = −1.57, p = .004). Participants with a family history of diabetes had significantly higher behaviour scores (B = 1.89, p = .020). History of foot ulcer emerged as the strongest predictor in the model (B = −5.60, p <.001), with individuals reporting previous ulcers demonstrating lower behaviour scores. Knowledge category independently predicted behaviour (B = 1.83, p = .041).

Age, marital status, occupation, type of diabetes, comorbidity, complications, and type of medications were not significantly associated with behaviour score. No evidence of multicollinearity was detected.

## 4 Discussion

In Ras Al Khaimah, 77.3% of 384 adults with diabetes who attended diabetes services had poor scores on the eight-item knowledge scale, and only 4.7% had good scores on the Nottingham Assessment of Functional Foot Care (NAFF), with 69.3% moderate and 26.0% poor. Knowledge and behaviour categories were weakly associated (χ 2 (2) = 9.784, p = 0.008; Cramér’s V = 0.160.

In the adjusted logistic regression model, female gender was associated with lower odds of good knowledge (AOR = 0.45, 95% CI: 0.23–0.90, p = 0.023; reference: male), and participants with type 2 diabetes had lower odds of good knowledge compared with those with type 1 diabetes (AOR = 0.43, 95% CI: 0.21–0.87, p = 0.020). Five were independently associated with the NAFF score in the adjusted linear model: female gender (B = 3.80, 95% CI 1.99 to 5.61, p < 0.001), history of foot ulcer (B = −5.60, 95% CI −7.81 to −3.38, p < 0.001), educational level (B = −1.57, 95% CI −2.64 to −0.51, p = 0.004), family history of diabetes (B = 1.89, 95% CI 0.30 to 3.48, p = 0.020) and good knowledge (B = 1.83, 95% CI 0.07 to 3.60, p = 0.041; reference poor knowledge). Both models accounted for a modest share of variance (Nagelkerke R² = 0.137; adjusted R² = 0.130), so most variability in knowledge and behaviour was not explained by the variables measured. As all measures were obtained at a single point in time, the findings below describe associations only and do not establish that any variable causes or precedes another.

The proportion classified as having poor knowledge was higher than in comparable Gulf primary care surveys: 47% among 488 adults with type 2 diabetes in Dubai, while 79.3% in Kuwait and approximately 70% in Riyadh were classified as adequately knowledgeable, and the mean score in Bahrain was about 73% of the maximum (14–17). These comparisons are indicative rather than direct, since the studies used different instruments and different cut-points, and the threshold alone can shift the reported prevalence of poor knowledge substantially. A closely similar estimate was reported in rural south India, where 70.1% had poor foot care knowledge (18).

Item-level responses (**Figure 1**) indicate that the low overall scores were driven largely by the four items assessing exposure to formal education rather than by conceptual awareness. Between 61.7% and 70.1% of participants answered the four content items correctly, whereas only 22.7% had attended a foot care class, 26.3% had read a foot care handout, 26.7% a handout on footwear, and 23.2% possessed any foot care material. Because the threshold for good knowledge (≥6 of 8) cannot be reached without affirmative responses to at least two exposure items, the figure of 77.3% reflects limited contact with structured education as much as limited factual awareness. This is consistent with the Dubai data, where awareness was higher among those who had received information from the Dubai Diabetes Centre (66%) than among those who had not (15).

Reported behaviour was predominantly moderate (69.3%), and good practice was uncommon (4.7%) — lower than in Dubai (28%) and Kuwait (30.8%), although differing instruments and cut-points mean the gap cannot be attributed to practice alone (14, 15). What is consistent across these settings, and in studies from Brazil, India and Bangladesh (19, 20), is that good practice is considerably less common than good knowledge, and that the practices most often omitted are those requiring specific technique rather than general adherence: in Dubai, avoiding moisturizer between the toes had the lowest practice (38%) against 91% for medication adherence, and in Riyadh only 31.4% dried carefully between the toes despite 70% being classified as knowledgeable (15, 17).

The knowledge–behaviour association was weak in both analyses that examined it. Cross-tabulation yielded Cramér’s V = 0.160, and in the adjusted model the good knowledge category corresponded to a 1.83-point higher NAFF score (95% CI 0.07 to 3.60) on a scale with an observed standard deviation of 7.54 — approximately one quarter of a standard deviation, with a lower confidence limit close to zero. The estimate is therefore small and imprecise. A comparable pattern is evident in Kuwait, where 79.3% had good knowledge but only 30.8% good practice [15], and in Riyadh (17). The Health Belief Model and Social Cognitive Theory offer one account of why knowledge may be insufficient on its own, proposing that action also depends on perceived susceptibility, perceived barriers, self-efficacy and environmental facilitators (21, 22); none of these constructs was measured here, so this is offered as a hypothesis for future testing rather than as a finding of this survey.

Women had lower odds of good knowledge than men, while type of diabetes was also associated with knowledge, with participants with type 2 diabetes showing lower odds of good knowledge than those with type 1 diabetes. Educational status was significant only at the overall variable level, and the individual contrasts were imprecise because of the small number of participants in the illiterate reference category. Occupation was not significantly associated with knowledge. These findings should therefore be interpreted cautiously, and the mechanisms underlying the observed differences cannot be established from the present cross-sectional data.

The variables associated with behaviour differed from those associated with knowledge. The two models are not directly comparable - they have different outcomes (a binary category versus a continuous score) and specify some variables differently- so a variable significant in one and not the other should not be read as an internal inconsistency. Women scored higher than men on the NAFF (B = 3.80, 95% CI 1.99 to 5.61), about half a standard deviation, in the opposite direction to the gender association seen for knowledge and the Dubai finding of better practice among men [14]. Alternatively, the instruments may be measuring different constructs: the knowledge scale being weighted toward documented exposure to education, the NAFF toward routine self-care activities. We do not interpret the divergence as evidence about gender roles, and would need data on household roles and health service contact that were not collected to test this.

History of foot ulcer showed the largest association (B = −5.60, 95% CI −7.81 to −3.38), approximately three quarters of a standard deviation lower. As both variables were measured at the same time, there is no temporal order and at least three explanations are plausible. Functional or treatment-related constraints occurring after ulceration may limit the activities the NAFF scores; psychological sequelae such as diabetes distress may limit engagement; or barriers that played a role in ulceration may have endured. Distinguishing these requires longitudinal or qualitative data. Irrespective of mechanism, the association identifies people with previous ulceration as a group with measurably lower reported preventive care, which is clinically relevant given their recurrence risk (5, 11).

Education level was negatively associated with behaviour score (B = −1.57, 95% CI −2.64 to −0.51) when entered as a single ordered term, assuming an approximately constant change across adjacent categories, but this assumption was not formally tested. Education was not clearly associated with knowledge in the same sample. The result also differs from reports from Bahrain (16) and Saudi Arabia, and whether this reflects local factors, the ordinal specification or residual confounding cannot be determined here (8, 23). Family history of diabetes was associated with a higher score (B = 1.89, 95% CI 0.30 to 3.48), though the lower confidence limit is close to zero. Age, marital status, occupation, type of diabetes, comorbidity, complications and medication type were not associated with behaviour score.

The k-means analysis was exploratory and descriptive, undertaken to characterize how preventive practices co-occur rather than to test a hypothesis. It identified low adherence (44.8%), low–moderate adherence (23.7%) and high adherence (31.5%) profiles. Mean behaviour scores differed across them (F(2, 381) = 116.81, p < 0.001), but this follows from the clustering procedure itself and is not an independent result. The association between cluster and knowledge was weak and non-monotonic (χ²(2) = 6.42, p = 0.040; Cramér’s V = 0.129): good knowledge was most frequent in the low-adherence cluster (26.7%), intermediate in the high-adherence cluster (24.0%) and least frequent in the low–moderate cluster (13.2%). Given the small effect size and absence of an ordered gradient, this should not be read as evidence that knowledge is unrelated or inversely related to behaviour; the adjusted regression is the more appropriate test and showed a small positive association. The clusters were derived from this sample without stability testing or independent replication, and their value is descriptive: low adherence was the most common profile and preventive practices were unevenly distributed.

### 4.1 Clinical and public health implications

Three implications follow from the estimates obtained. First, exposure to structured foot care education was low, with fewer than one quarter of participants having attended a class or received written materials -a directly modifiable service-level gap and a reasonable first target locally. Second, because the knowledge–behaviour association was small, education delivered in isolation would not be expected to produce large improvements in practice, and multicomponent programmes addressing barriers, skills and follow-up are more likely to be effective, though this expectation requires local testing. Third, participants with previous foot ulceration reported the lowest preventive care of any subgroup examined and warrant structured review rather than standard education alone (5, 11). The associations with gender, occupation and educational level identify groups in which knowledge or reported practice was lower and may be useful for targeting, but they do not indicate why these differences arise, and intervention content should not be built around unverified mechanisms.

### 4.2 Strengths, limitations and recommendations for future research

This study addresses an important and under-researched area by assessing foot self-care knowledge and behaviours among adults with diabetes mellitus in Ras Al Khaimah, UAE, thereby contributing local evidence to the literature. A further strength was the use of structured and previously developed instruments, including the eight-item foot-care knowledge questionnaire and the Nottingham Assessment of Functional Foot Care (NAFF), with content validity and reliability assessed for the study questionnaire. The inclusion of participants from both primary and secondary healthcare settings also provided information from different levels of diabetes care.

Several limitations should be considered when interpreting the findings. First, the cross-sectional design precludes establishing temporal or causal relationships between foot-care knowledge and behaviours. Second, the use of convenience sampling may have introduced selection bias and limits the generalizability of the findings to adults with diabetes in other healthcare settings or regions of the UAE. Third, reliance on interviewer-administered self-reported data may have introduced recall and social desirability biases, potentially affecting the accuracy of reported foot self-care behaviours. Fourth, although several sociodemographic and clinical variables were assessed, potentially relevant psychosocial and contextual factors, such as self-efficacy, health beliefs, access to foot-care resources, and environmental barriers, were not measured and may have contributed to residual confounding. Finally, the study was conducted in selected healthcare facilities in Ras Al Khaimah; therefore, the findings should be generalized cautiously to other populations and healthcare systems.

Future research could use longitudinal or prospective designs to examine temporal relationships between foot-care knowledge and behaviours. Qualitative studies may further explore cultural, psychosocial, and contextual factors influencing foot self-care. Intervention studies evaluating tailored, multicomponent educational and behavioural programmes are also warranted to determine whether improving knowledge and addressing identified barriers can improve foot self-care behaviours and reduce diabetic foot complications.

## 5 Conclusions

The study identified significant gaps in foot self-care knowledge and behaviours among adults with diabetes in Ras Al Khaimah, with most participants demonstrating poor knowledge and moderate to poor self-care practices. Factors such as gender, occupation, educational level, family history of diabetes, and history of foot ulcers were associated with variations in knowledge and behaviour. These findings highlight the need for targeted, multifaceted educational interventions and support strategies tailored to the local context to enhance foot care and reduce the risk of diabetic foot complications in this population.

## Data Availability

The original contributions presented in the study are included in the article/supplementary material. Further inquiries can be directed to the corresponding authors.
